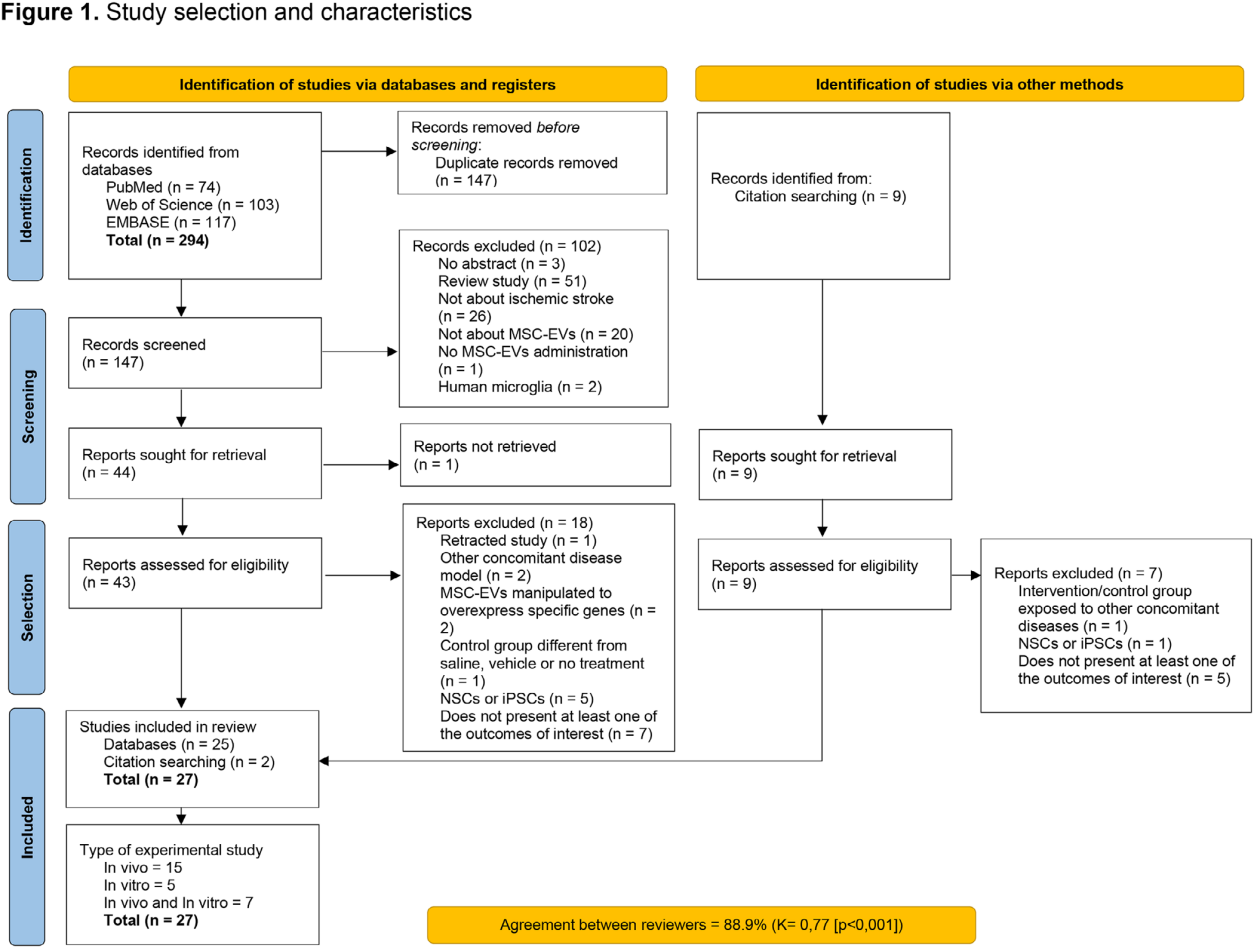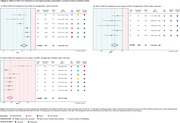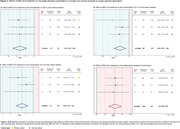# Extracellular Vesicles Treatment Induces Pro‐resolutive Microglial Changes in Experimental Models of Ischemic Stroke: A Systematic Review and Meta‐analysis

**DOI:** 10.1002/alz.091964

**Published:** 2025-01-03

**Authors:** Luis Pedro Bernardi, Thomas Hugentobler Schlickmann, Giovanna Carello‐Collar, Francieli Rohden, Diogo O. Souza, Eduardo R. Zimmer

**Affiliations:** ^1^ Universidade Federal do Rio Grande do Sul, Porto Alegre, Rio Grande do Sul Brazil; ^2^ Universidade Federal do Rio Grande do Sul, Porto Alegre, RS Brazil; ^3^ Universidade Federal do Rio Grande do Sul, Porto Alegre Brazil; ^4^ McGill University, Montreal, QC Canada; ^5^ Brain Institute of Rio Grande do Sul ‐ Pontifícia Universidade Católica do Rio Grande do Sul, Porto Alegre, Rio Grande do Sul Brazil

## Abstract

**Background:**

Ischemic stroke (IS) is a risk factor for developing Alzheimer’s disease (AD). In this context, microglial activation is a shared cellular response to these two conditions that can be either beneficial or detrimental. Previous research has established that mesenchymal stem cell‐derived extracellular vesicles (MSC‐EVs) treatment leads to enhanced functional recovery and reduced brain infarct volume in animal IS models. However, current literature findings are unclear when addressing the effects of MSC‐EVs treatment on microglial activation. Thus, we aimed to investigate how MSC‐EVs treatment alters microglial activation parameters in IS models.

**Method:**

The protocol of this systematic review was registered in PROSPERO (CRD42023463152) and followed the PRISMA 2020 statement. We searched EMBASE, PubMed, and Web of Science from database inception to October 2023 for studies with animal or cell culture IS models using MSC‐EVs as intervention and measuring microglial activation outcomes compared to control. We performed a random‐effects meta‐analysis using standardized mean differences (SMD) as the effect measure with the metafor and metaviz packages in R (v4.2.1) (FDR‐adjusted p‐value<0.05).

**Result:**

The database search identified 294 records, from which 27 were included (Fig. 1). Meta‐analysis showed that *in vivo* MSC‐EVs treatment resulted in a lower number of total microglia (Iba1+ cells) (SMD = ‐1.45 [‐2.19,‐0.71 95%CI] p<0.001, Fig. 2A) and CD16+ microglia (SMD = ‐1.84 [‐2.62,‐1.05 95%CI] p<0.001, Fig. 2B), but in a higher number of CD206+ microglia (SMD = 1.95 [1.01,2.88 95%CI] p<0.001, Fig. 2C) in the brain across different IS models and age groups. In microglia cell cultures submitted to oxygen‐glucose deprivation, MSC‐EVs treatment decreased the levels of TNF‐α (SMD = ‐3.32 [‐4.95,‐1.69 95%CI] p<0.001, Fig. 3A), IL‐1β (SMD = ‐3.45 [‐5.57,‐1.32 95%CI] p<0.001, Fig. 3B), and IL‐6 (SMD = ‐2.95 [‐4.50,‐1.40 95%CI] p<0.001, Fig. 3C) in the culture medium, while increasing the gene expression levels of Arg‐1 (SMD = 5.41[3.54,7.29 95%CI] p<0.001, Fig. 3D).

**Conclusion:**

We demonstrated that MSC‐EVs treatment in IS models reduces the expression of pro‐inflammatory microglial activation markers (CD16) and cytokines (TNF‐α, IL‐1β, IL‐6) while increasing the expression of anti‐inflammatory markers (CD206, Arg‐1). Our results suggest that MSC‐EVs treatment modulates microglia towards a pro‐resolutive state, potentially contributing to the recovery of damaged brain tissue in IS and, consequently, in AD.